# The role of face regions in remote photoplethysmography for contactless heart rate monitoring

**DOI:** 10.1038/s41746-025-01814-9

**Published:** 2025-07-26

**Authors:** Maksym Bondarenko, Carlo Menon, Mohamed Elgendi

**Affiliations:** 1 Biomedical and Mobile Health Technology Research Lab, ETH Zürich, 8008 Zürich, Switzerland; 2 School of Computation, Information and Technology (CIT), Department of Computer Science, Technical University of Munich (TUM), 80333 Munich, Germany; 3 Department of Biomedical Engineering and Biotechnology, Khalifa University of Science and Technology, Abu Dhabi P.O. Box 127788, Khalifa, United Arab Emirates

**Keywords:** Biomarkers, Engineering

## Abstract

Heart rate (HR) estimation is crucial for early cardiovascular diagnosis, continuous monitoring, and various health applications. While electrocardiography (ECG) remains the gold standard, its discomfort and impracticality for continuous use have spurred the development of non-contact methods like remote photoplethysmography (rPPG). This systematic review (PROSPERO: CRD 42024592157) examines 70 studies to assess the impact of Region of Interest (ROI) selection on HR estimation accuracy. Most methods (36.8%) use the holistic face, while forehead and cheek areas (24.5% and 21.7%) show superior accuracy. Machine learning-based approaches outperform traditional methods under motion artifacts and poor lighting, achieving Mean Absolute Error and Root Mean Square Error below 1.0 for some datasets. Combining multiple patches improves performance, though increasing ROIs beyond 60 patches results in diminishing returns and higher computational complexity. These findings highlight the significance of ROI optimization for robust rPPG-based HR estimation.

## Introduction

Heart rate (HR) and blood pressure (BP) estimation are essential for early detection of cardiovascular diseases, continuous health monitoring, emotion detection, and assessing other vital parameters. Although electrocardiography (ECG) is the gold standard for HR detection due to its high accuracy, it has notable drawbacks, including high equipment costs, user discomfort, and challenges with continuous, everyday monitoring. To address these limitations, photoplethysmography (PPG) was developed, which accurately measures HR by detecting light reflected from a wearable device, such as an oximeter. Traditional PPG still relies on contact-based devices, such as finger sensors and wearables, which, while effective in controlled environments, present significant limitations in real-world applications. These devices can cause discomfort during prolonged use and may fail in scenarios involving delicate or compromised skin conditions, such as burns, eczema, or post-surgical recovery. Furthermore, populations such as neonates, elderly patients, and individuals in intensive care units often face additional risks or challenges with contact-based monitoring. In neonates, especially those in intensive care, traditional contact-based heart rate monitoring can be problematic due to their delicate skin and the risk of injury. Studies have explored alternative methods, such as forehead monitoring of heart rate in neonatal intensive care, to address these challenges^1^. In these contexts, remote photoplethysmography (rPPG) offers a transformative solution, enabling non-invasive and continuous heart rate monitoring using standard cameras, which could significantly enhance healthcare delivery in both clinical and at-home settings.

The rPPG was introduced to overcome these challenges. Unlike traditional PPG, rPPG estimates HR using standard RGB cameras by detecting subtle light changes on a subject’s face, eliminating the need for direct physical contact, however bringing comparable results^2^. The most effective regions for capturing these skin color changes are areas with lower skin density, such as the cheeks and forehead^3,4^. Despite its advantages, rPPG faces several challenges, including sensitivity to varying lighting conditions, movement artifacts, false face detection, and obstructions caused by masks, hair, or clothing. To filter noisy signals, a Signal Quality Index (SQI) was introduced, with a threshold value *N*_SQI_ < 0.293^5^. Achieving optimal accuracy in rPPG also requires maintaining a stable position, an appropriate distance from the camera, and minimizing motion.

Early traditional rPPG methods, such as Principal Component Analysis (PCA)^6^, Independent Component Analysis (ICA)^7^, the chrominance-based CHROM^8^ method, the GREEN channel^9^ approach, and the Plane-Orthogonal-to-Skin (POS)^10^ algorithm, focused primarily on noise reduction. These methods typically began by detecting the face and defining a Region of Interest (ROI) based on empirical knowledge, followed by extracting pulse signals from the RGB channels within the ROIs. HR was then derived from the signals using techniques like Discrete Fourier Transform (DFT) and peak detection.

With the rise of machine learning (ML), new approaches^11^ have emerged that utilize neural network models, particularly convolutional neural networks (CNNs), to extract spatiotemporal features from facial videos. Examples include Dual-Gan^12^, TranPulse^13^, and MAR-rPPG^14^. These methods aim to develop robust end-to-end network structures capable of accurately estimating physiological parameters under real-world conditions by enhancing temporal correlations and minimizing redundant information. Unlike traditional approaches, which rely on handcrafted features derived from skin texture, rPPG signals, and manually defined ROIs, ML methods improve HR estimation quality and significantly reduce reliance on clean input data. On the other hand, ML-based methods require labeled data for training and are more computationally difficult, when the traditional methods could be efficient and low-complexity^15^. Apart from HR and BP^16^ an estimation of HRV (Heart Rate Variability), SpO2 (Blood Oxygen Saturation) and anxiety detection^17^ are possible. In this paper, we conduct a systematic review of 70 studies from the past decade, assessing different algorithms for HR estimation. We examine 20 commonly used datasets and evaluate the impact of ROI selection on HR estimation through rPPG.

**Fig. 1:**
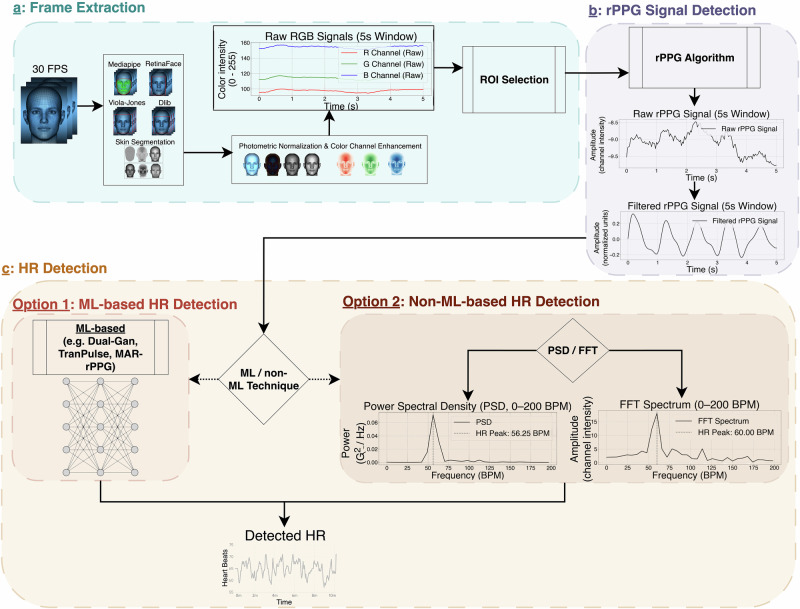
Flow diagram of rPPG estimation steps. **a** Face Detection algorithms with Color Channel Enhancement and Photometric Normalization (Illumination Equalization, Color Normalization)—the light-blue area with a dashed border; followed by ROI Selection methods and Pre-Processing (Bandpass Filter, Baseline Correction, Signal Filtering Gaussian/Median) for (**b**) rPPG Signal detection and filtering—the light-purple area with a dashed border. Next, (**c**) HR detection—the light-yellow area with a dashed border. Either using ML-based methods(Option 1: the light-red area with a dashed border) with labeled data to estimate HR, or using traditional methods (Option 2: the light-brown area with dashed border) without training data for HR prediction, e.g., via PSD or FFT.

**Fig. 2:**
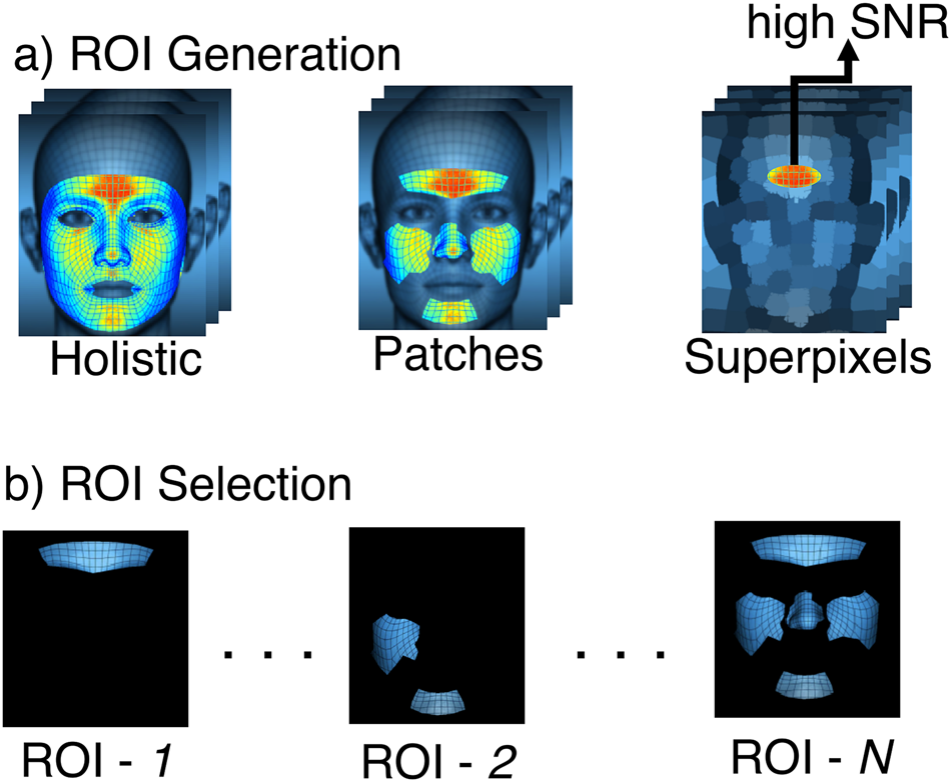
Example of ROI Generation algorithms. **a** Using whole (holistic) face, separate facial patches (regions), and superpixel techniques (segmentation of the image into smaller, meaningful regions based on pixel similarity). Later, (**b**) single, multiple or set of combinations of sub-ROIs can be used.

Figures 1 and 2 provide a synthesized flow diagram of the typical remote photoplethysmography processing pipeline, based on our literature review. This chart presents a high-level overview of commonly employed rPPG-based physiological monitoring stages. While this is a generalized representation that may not capture every method in its entirety, it highlights critical steps within the rPPG pipeline. The flow diagram emphasizes the importance of ROI segmentation, a critical step in rPPG, as it significantly impacts the quality and reliability of the extracted signal. ROIs typically include facial areas with high blood perfusion, such as the forehead and cheeks regions, to provide more stable rPPG signals. Common techniques such as superpixel segmentation, skin segmentation, and triangulation help isolate relevant skin areas and enhance spatial smoothness during data processing.

After ROI segmentation, the flow chart illustrates several pre-processing steps commonly used to enhance signal quality, including color channel selection, photometric normalization, and signal filtering. These steps help mitigate noise from environmental factors (e.g., lighting changes) and movement artifacts. The flow diagram also differentiates between traditional methods (e.g., CHROM, ICA, PCA, GREEN) and ML-based approaches, providing a snapshot of the variety of available techniques for physiological signal extraction.

Traditional methods, such as Principal Component Analysis (PCA), Independent Component Analysis (ICA), and Fast Fourier Transform (FFT), rely on predefined algorithms and handcrafted features, making them computationally efficient and cost-effective for consumer devices. However, these methods often struggle with noise and variability in real-world conditions. ML-based methods, such as convolutional neural networks (CNNs), automatically learn complex spatiotemporal patterns from data, excelling in challenging scenarios involving motion artifacts or varying lighting. Despite their accuracy, ML approaches are computationally intensive, requiring substantial resources for training and deployment, which may increase costs for healthcare systems and limit their scalability in consumer-grade devices. Hybrid approaches that blend the efficiency of traditional methods with ML’s adaptability could offer a promising middle ground.

Furthermore, contact-based PPG devices, while effective in controlled settings, often show reduced performance across diverse skin tones due to varying melanin levels that affect light absorption and reflection. Additionally, conditions that induce peripheral vasoconstriction, such as hypothermia or cardiovascular diseases, can impair signal quality by reducing blood flow in extremities^18^. In contrast, remote PPG (rPPG) leverages facial regions with relatively stable blood perfusion, such as the forehead and cheeks, making it less affected by these limitations and better suited for diverse populations and clinical conditions.

Overall, this flow diagram does not represent a comprehensive, step-by-step process but rather serves as a conceptual overview of the rPPG processing pipeline, with an emphasis on ROI selection, pre-processing techniques, and physiological parameter estimation.

## Results

### Publications

**Fig. 3:**
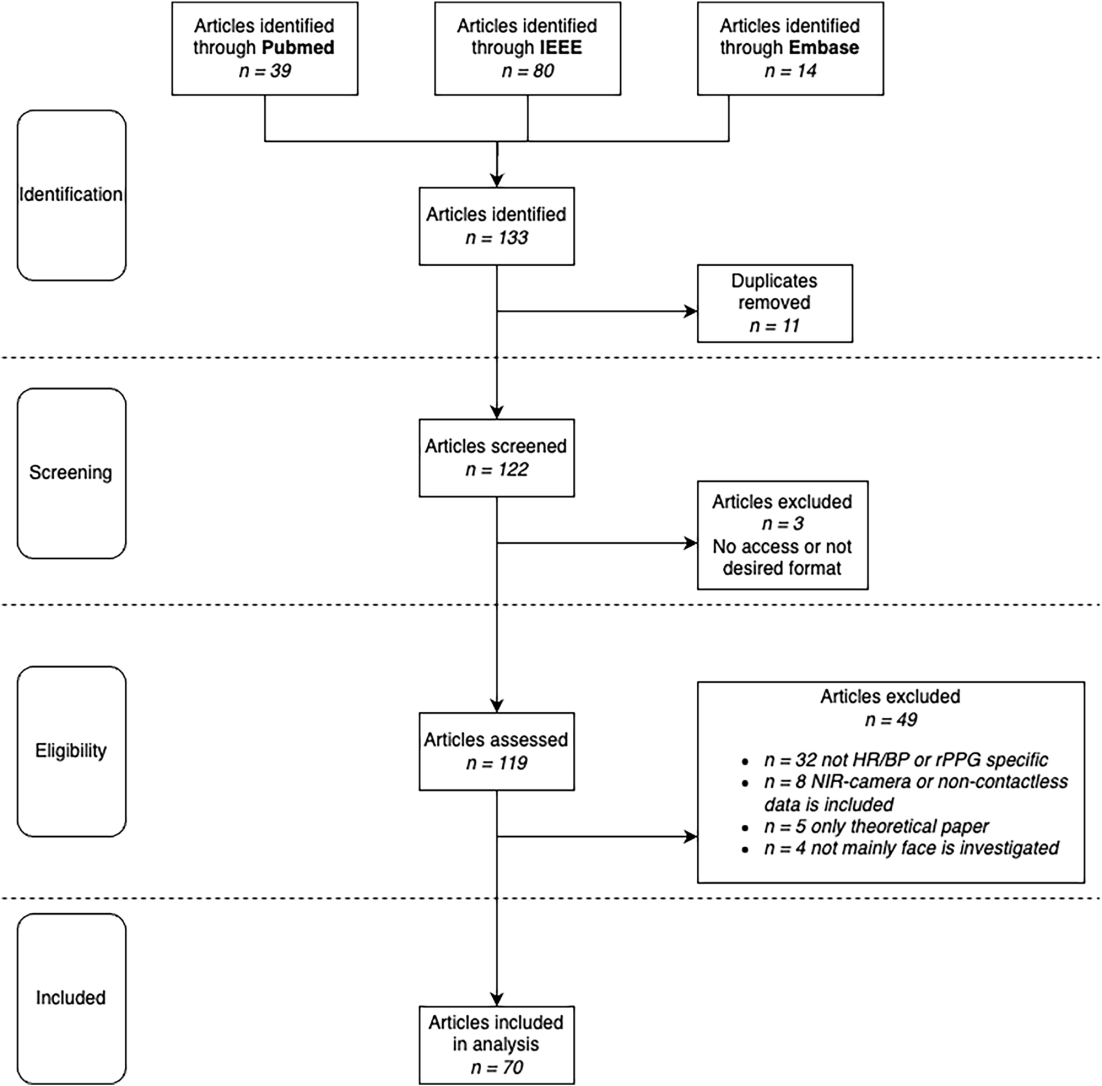
Search workflow with the identification, screening, eligibility, and inclusion phases in the review according to PRISMA guidelines. Out of 133 initially identified papers, 70 were finally included in the systematic literature review.

This literature review identified a total of 70 studies evaluating various algorithms for heart rate estimation using remote photoplethysmography (rPPG), as illustrated in Fig. 3. The search yielded 39 articles from PubMed, 80 from IEEE Xplore, and 14 from Embase. After an initial screening, 11 duplicate studies and 3 articles that were inaccessible or incompatible were excluded. Of the remaining articles, 49 were deemed ineligible: 32 did not focus on HR or BP detection, 8 utilized additional technologies such as infrared or near-infrared (NIR) imaging or employed contact-based devices, 5 were theoretical papers, and 4 did not primarily collect data from facial regions. This selection process identified a notable trend: an increasing focus on ROI selection, with studies reporting optimal results for algorithms that incorporate multiple ROIs.

**Fig. 4:**
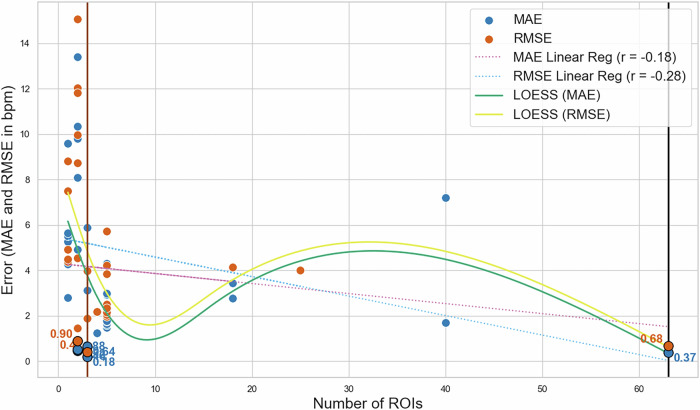
MAE and RMSE per ROIs with LOESS function on datasets COHFACE, MAHNOB-HCI and PURE. The best results with error < 1.00 are concentrated around 3 and 63 ROIs (also marked with vertical lines). A total of 33 values for MAE and 29 values for RMSE were used.

A notable trend observed in these studies is the increasing focus on ROI selection, with studies reporting optimal results for algorithms that incorporate multiple ROIs^19^ as shown in Fig. 4 with a total of 33 values for MAE and 29 values for RMSE. Table 1 highlights key variables influencing ROI performance, such as the predominance of participants with lighter skin tones and the geographic concentration of datasets. This lack of diversity underscores the need for future studies to incorporate participants with varied ethnicities and skin tones, particularly from underrepresented regions, to better generalize ROI-based HR estimation techniques. Another key observation is the shift towards machine learning (ML) techniques in HR estimation algorithms. ML-based approaches have shown enhanced accuracy, especially for challenging cases involving motion, using publicly available datasets. Recent top-performing methods^12,20,21^ commonly employ combinations of multiple facial ROIs, in contrast to earlier studies, which predominantly relied on single-region analysis.

**Table 1:** Overview of public datasets used for data validation in articles

Dataset name	Year	Number of subjects and their sex	Ethnicity or skin color	Age in years	Number of videos	Video length in seconds	Camera	FPS	Resolution in pixels	HR measurement device
UBFC-rPPG^53^	2017	42 (31 male, 11 female)	N/A	N/A	42	60 sec	Logitech C920 HD Pro	30 fps	640 × 480	CMS50E
PURE^54^	2014	10 (8 male, 2 female)	N/A	N/A	60	60	eco274CVGE	30	640 × 480	CMS50E
COHFACE^55^	2016	40 (28 male, 12 female)	N/A	N/A	160	60	Logitech HD C525	20	640 × 480	SA9308, SA9311M
MMSE-HR^66^	2016	40 (23 female, 17 male)	Fitzpatrick scale (II=8, III=11, IV=17, V+VI=4)	N/A	102	30-60	N/A	25	1040 × 1392	N/A
VIPL-HR^56^	2018	107 (79 male, 28 female))	N/A	N/A	2378	30	Logitech C310, RealSense F200, HUAWEI P9	25, 30, 30	960 × 720, 1920 × 1080, 1920 × 1080	CMS60C
LGI-PPGI^67^	2018	25 (20 male, 5 female)	majority is Caucasian	25-42	100	120	Logitech HD C270	25	N/A	CMS50E
MAHNOB-HCI^57^	2011	27	N/A	N/A	527	60-180	Allied Vision Stingray F-046C, F-046B	61	780 × 580	N/A
MR-NIRP (auto)^68^	2020	18 (16 male, 2 female)	Indian, Caucasian, Asian	20-60	190	120	FLIR Grasshopper3 GS3-PGE-23S6C-C	30	640 × 640	CMS50D+
MR-NIRP (indoor)^69^	2020	8 (6 female, 2 male)	4 Indian, 3 Caucasian, 1 Asian	20-40	15	180	Point Gray Flea3 FL3-U3-13E4C-C and FLIR Blackfly BFLY-U3-23S6C-C	30	640 × 640	CMS50D+
PFF^70^	2017	13	N/A	N/A	24	180	Nikon D5300	50	1280 × 720	MIO Alpha II
IIP-F^71^	2021	26 (16 male, 10 female)	N/A	N/A	26	N/A	Microsoft Lifecam Studio	30	N/A	N/A
IIP-W^71^	2021	26 (16 male, 10 female)	N/A	N/A	26	N/A	wearable Aoni A36 webcam	25	N/A	N/A
Self-rPPG^72^	2022	13 (10 male, 3 female)	N/A	N/A	78	60	N/A	30	640 × 480	CMS50E
OBF^73^	2018	100 (61 male, 39 female)	Caucasian, Asian, Others	16-68	200	300	Blackmagic URFA mini	60	1920 × 1080	NX-EXG2B
UBFC-Phys^74^	2021	56 (46 female, 10 male)	N/A	19-38	56	180	EO-23121C RGB	35	1024 × 1024	Empatica E4
BIDMC^52^,^75^	2018	51 (32 female, 19 male)	N/A	19-90+	53	480	N/A	N/A	N/A	N/A
BP4D+^76^	2016	140 (82 female, 58 male)	Black, White, Asian, Hispanic/Latino, Native American, others	18-66	N/A	N/A	N/A	N/A	N/A	N/A
TokyoTech^77^	2019	9 (8 male, 1 female)	N/A	20-60	27	180	own prototype of RGB-NIR Camera	30	640 × 480	Procomp Infinity T7500M
MMPD^78^	2023	33	Fitzpatrick skin types 3-6	N/A	660	60	Samsung Galaxy S22 Ultra	30	1280 × 720	HKG-07C+
rPPG^79^	2019	8 (7 male, 1 female)	Fitzpatrick skin types 1-4	24-37	52	60-80	Logitech C920, Microsoft VX800, Lenovo B590	15	1920 × 1080, 640 × 480, 640 × 480	Choicemmed MD300C318

Detailed information regarding camera, ground-truth devices and subjects is included.

### Characteristics of participants in datasets

Most of the reviewed studies utilize publicly available datasets, with 20 studies^22–41^ relying exclusively on in-house datasets for testing, and 8 studies^42–49^ combining both public and in-house datasets. Two studies^50,51^ do not specify details about the testing subjects. The datasets predominantly feature healthy male and female participants, typically aged 18 to 60. Most videos in these datasets are 5 minutes or shorter, though the BIDMC dataset^52^ includes videos up to 8 minutes. Video resolutions range from 1920 × 1080 to 640 × 480 pixels, and filming conditions vary widely in terms of subject movement, head rotations, physical activities, and lighting conditions.

The most frequently used datasets are UBFC-rPPG^53^, PURE^54^, COHFACE^55^, VIPL-HR^56^, and MAHNOB-HCI^57^, with 27, 24, 13, 11, and 6 mentions, respectively. UBFC-rPPG demonstrates relatively low error rates due to its steady setup, with controlled indoor illumination and variable sunlight exposure, as subjects engage in a quiz. The PURE dataset incorporates six different scenarios: steady, talking, slow translation, fast translation, slow rotation, and medium rotation. VIPL-HR generally reports higher error rates, likely due to its nine distinct conditions that include various head movements and lighting conditions, making it a valuable benchmark for assessing algorithm stability. It is important to note that most datasets, whether publicly available or in-house, contain a larger proportion of male subjects compared to female subjects. This gender imbalance may introduce biases and reduce accuracy in real-world applications.

### Types of camera devices and reference systems

All dataset videos are recorded using standard RGB cameras (non-IR/NIR), with ground-truth HR measurements provided by finger pulse oximeters or wrist-worn devices. Table 1 includes detailed information on the specific filming settings and reference devices used across studies. It highlights critical variables relevant to ROI selection, including skin color, ethnicity, and geographic distribution of participants. Notably, most datasets either lack explicit reporting of participants’ skin tones or primarily include lighter-skinned individuals. This limitation can bias the effectiveness of ROI-based HR estimation, as skin color significantly impacts light absorption and reflection, which are critical for rPPG signal quality. Expanding datasets to include participants from diverse ethnic backgrounds and geographic locations is essential to better understand how ROI performance varies across skin tones, lighting conditions, and cultural contexts. Such efforts would ensure the development of rPPG technologies that are robust and equitable across global populations.

### ROIs used for HR estimation

Multiple studies emphasize the critical role of Region of Interest (ROI) selection in the accuracy of heart rate (HR) estimation, noting that inappropriate ROI choices can introduce significant errors. Kwon et al.^58^ divided the face into seven regions to evaluate each for signal quality, while Poh et al.^59^ recommended an ROI that spans 60% of the full face’s width and its entire height. Later, Zhao et al.^60^ focused on an ROI below the eye line, covering skin areas around the nose, mouth, and cheeks.

According to Dae-Yeol Kim et al.^4^, facial areas with larger surface areas and thinner skin, such as the cheeks and forehead, tend to yield more reliable results and are less affected by light reflections. In contrast, the nose area is considered less reliable for capturing skin color changes. Kim et al. proposed five specific facial regions (TOP-5) characterized by lower variability and noise, with an average skin thickness of 1191.11 *μ*m and a pixel count of 2431. Detailed information regarding ROIs, their size, skin thickness and visual representation is shown in Table 2. According to Kim et al.^4^, using those facial patches improves the MAE on the UBFC dataset for POS and CHROM methods from 1,87 to 1,85 and from 2.67 to 1.5 accordingly. Similarly, MAE for PPGI dataset is improved from 4.04 to 3.61 for POS and from 4.04 to 2.93 for CHROM methods.

**Table 2:** Details of ROIs, including sampled pixels, thickness, image representation, and common co-included regions

ROI Name	Pixels Sampled	Thickness (*μ*m)	ROI Image	Common Co-Included ROIs
Upper Medial Forehead	504	1245.63		Whole Forehead (Forehead and Glabella)
Lower Medial Forehead	454	1221.88		
Glabella	775	1386.11		Whole Forehead (Forehead and Glabella)
Right Malar	794	1086.20		Left Malar (Cheek)
Left Malar	955	1086.20		Right Malar (Cheek)

The TOP-5 regions were taken, according to Li et al.^61^ and Kim et al.^4^.

Similarly, Li et al.^61^ analyzed 28 facial regions defined anatomically and identified the most effective ROIs: the glabella, medial forehead, left and right lateral forehead, left and right malar regions, and upper nasal dorsum. Among these, the glabella demonstrated the best overall performance across both motion and cognitive datasets.

Furthermore, due to the symmetry of cheek areas, it might be more robust to the noise and can enable dynamic substitution of ROIs if some part of the face is obscured due to the head rotation^62^. Furthermore, the forehead and cheeks have a larger flat area, which has a positive impact on the Signal-to-noise ratio (SNR). Apart from that, there could be benefits of using those areas, as they are mostly free of facial hair, accessories and facial expression change.

**Fig. 5:**
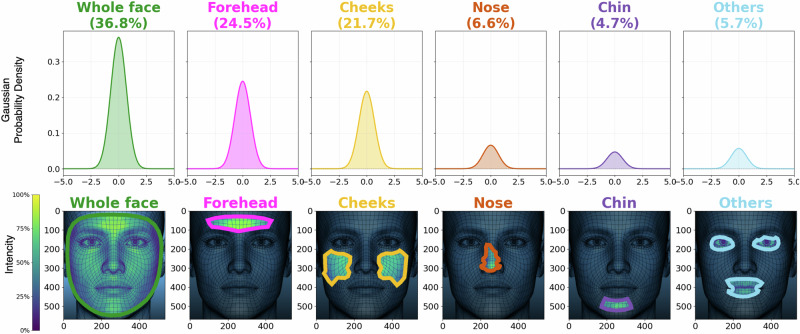
Most commonly analyzed regions of interest (ROIs) in reviewed studies and their pixel intensity characteristics. The top row illustrates the percentage of studies utilizing specific ROIs, including the whole face (36.8%), forehead (24.5%), cheeks (21.7%), nose (6.6%), chin (4.7%), and other regions (5.7%). Gaussian probability density plots show the normalized intensity distributions within each ROI across studies. The bottom row depicts the spatial intensity maps (viridis colormap) of pixels within each ROI, highlighting areas of high and low intensity. Asymmetric ROIs, such as those from the nose and eyes, are shaped by the Mediapipe algorithm, which uses triangular regions that may reduce accuracy. Environmental factors, such as lighting, reflections, and head rotation, also impact ROI selection algorithms.

In real-world applications, certain facial regions may be obscured by facial hair, accessories, shadows, or masks, necessitating more precise ROI selection techniques. Some studies^37,63,64^ have adopted superpixel segmentation to isolate facial ROIs, excluding non-skin regions such as the mouth and eyes. This technique allows for the segmentation of skin areas with irregular shapes, unlike block-based segmentation. As shown in Fig. 5, the forehead and cheeks are the most frequently selected regions, used 26 and 23 times, respectively. The nose and chin appear less frequently, in 7 and 5 articles, respectively. However, a majority of studies (39) utilize the entire holistic face. Yaran Duan et al.^62^ introduced a self-adaptive ROI pre-tracking and signal selection method to mitigate motion artifacts using 18 facial patches. The ROIs are continuously tracked, with their visibility dynamically assessed based on the motion state. Similarly,^24^ implemented a symmetry substitution approach, where data from visible areas of the left and right cheeks are symmetrically replicated when facial rotation (30-45 degrees) obscures certain regions.

**Fig. 6:**
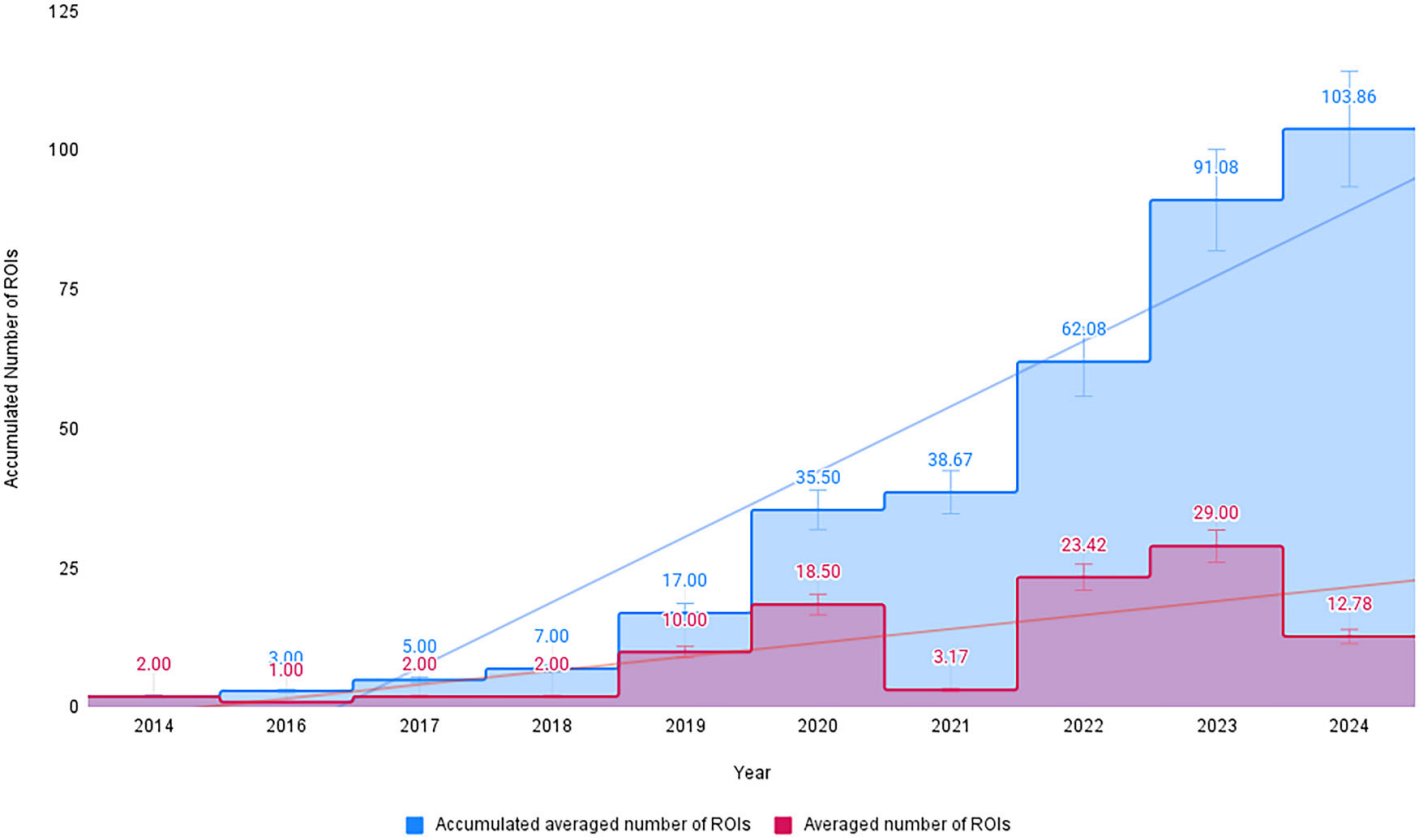
Accumulated averaged number of ROIs per Paper per Year. There is a notable trend of increasing amount of papers starting from the year 2019.

Haoyuan Gao et al.^19^ recommend an optimal range of 30 to 40 triangular ROIs on the face, cautioning that an excessive number of ROIs may lead the model to behave more as a face detection system than as an HR estimation tool. In their study, they utilized Delaunay triangulation to create 898 triangular ROIs. Recent works^19–21,43^ favor using multiple ROI combinations rather than averaging values from facial patches. Figure 6 illustrates the increasing trend in the number of ROIs used per study, which correlates with improved performance evaluations. This suggests that employing a larger number of facial ROI combinations can enhance algorithmic accuracy, though further data is needed for more robust statistics.

Because this work is a systematic literature review rather than an experimental study, we did not implement a common rPPG-to-HR extraction pipeline across datasets. Instead, we summarized the published performance that each primary study achieved with its own signal processing chain and then isolated the variable of interest, the number, and arrangement of ROIs, when comparing results. Consequently, absolute error values still reflect the underlying algorithmic diversity (e.g., CHROME, POS, Dual-GAN, etc.), but the review keeps the discussion centered on how ROI granularity modulates those outcomes. Future benchmark work in which identical extraction code is reapplied to every ROI configuration would be valuable, yet it lies beyond the scope of the present survey, whose aim is to map current practice rather than reimplement it.

### Performance evaluation

Figure 4 shows a trend toward an increasing number of ROIs across studies, along with improved Mean Absolute Error (MAE) and Root Mean Square Error (RMSE) values on datasets such as PURE^54^, COHFACE^55^, and UBFC-rPPG^53^. To capture non-linear data trends, we included a LOESS (Locally Estimated Scatterplot Smoothing) function. Unlike linear regression, LOESS fits a series of localized regressions across subsets of the data, generating a smooth curve that adapts to pattern changes, thus providing a more accurate visualization of the relationship between ROI quantity and error metrics (MAE and RMSE). The Pearson Correlation Coefficient (PCC) between the number of ROIs and error shows a moderate negative linear relationship, calculated as follows:

1 $${r}_{xy}=\frac{\mathop{\sum }\nolimits_{i = 1}^{n}({x}_{i}-\bar{x})({y}_{i}-\bar{y})}{\sqrt{\mathop{\sum }\nolimits_{i = 1}^{n}{({x}_{i}-\bar{x})}^{2}}\sqrt{\mathop{\sum }\nolimits_{i = 1}^{n}{({y}_{i}-\bar{y})}^{2}}}$$

where *x*_*i*_ and *y*_*i*_ are points at lag *i* of the rPPG and PPG signals, respectively. $\bar{x}$ and $\bar{y}$ represent their means. *N* is the number of points of the discrete signals.

Equations (2) Mean Absolute Error (MAE) and (3) Root Mean Square Error (RMSE) are commonly used metrics for evaluating error in models. are expressed as follows:

2 $$\,{\text{MAE}}\,=\frac{1}{N}\mathop{\sum }\limits_{i=1}^{N}| {y}_{i}-{x}_{i}|$$

3 $$\,{\text{RMSE}}\,=\sqrt{\frac{1}{N}\mathop{\sum }\nolimits_{i=1}^{N}{({x}_{i}-{y}_{i})}^{2}}$$

where *N* is the number of points and *x*_*i*_, *y*_*i*_ are the points at lag *i* of the rPPG and contact PPG signals, respectively.

From Figs. 4 and 6, it is evident that more recent, high-accuracy studies often use a larger number of smaller facial patches or combinations thereof. Taking more patches (e.g. more than 60) could slightly increase or even have the same performance but with much more computational effort. However, establishing a strong correlation between ROI quantity and error metrics (MAE/RMSE) remains challenging due to the limited number of studies with clearly documented ROI selection on public datasets. Additionally, as noted by Gao et al.^19^, an excessive number of ROIs can reduce accuracy. However, their analysis focused on the entire face rather than specific regions like the forehead and cheeks, which recent research has frequently emphasized.

As of October 2024, the best results on publicly available datasets, in terms of Mean Absolute Error (MAE) and Root Mean Square Error (RMSE), have been achieved by the following studies:

- Qian et al.^20^, utilized up to 64 distinct ROI patches (ROI combinations for noise reduction), distributed symmetrically across the face to ensure spatial diversity and enhance rPPG prediction. Introduced Spatial TokenLearner for identifying the most informative ROIs while suppressing noisy regions, and Temporal TokenLearner to mitigate disturbances such as motion artifacts and illumination changes.
- Zhao et al.^14^, Focused on precise ROI localization using attention regularization techniques. Improved robustness to motion artifacts and inconsistencies in ROI localization by utilizing MediaPipe, Masked Attention Regularization (MAR) and Enhanced rPPG Expert Aggregation (EREA).
- Hao Lu et al.^12^, proposed a Dual-GAN architecture to jointly model BVP predictors and noise distributions. Designed an ROI Alignment and Fusion (ROI-AF) block to align features across ROIs and address inconsistencies in noise distributions and used adversarial learning to disentangle BVP and noise components, enhancing robustness to environmental and physiological noise.
- Si-Qi Liu et al.^65^, introduced a lightweight spatiotemporal convolutional network (STConv) for rPPG estimation with a noise-disentangling module to separate environmental noise from physiological signals, guided by background features. Also, applied adaptive ROI selection for robustness across datasets and scenarios.
- Yaran Duan et al.^62^, divided the facial region into 18 small circular sub-ROIs, and grouped symmetrically into 9 main ROIs for robust tracking. Introduced a self-adaptive tracking system to discard occluded or noisy ROIs and retain their visible counterparts and applied spectral analysis to select the best-quality signal from available ROIs dynamically.

Table 3 provides specific MAE and RMSE values for the top five datasets. Overall, there is a clear trend toward employing a growing number of ROI combinations to improve heart rate estimation accuracy, particularly through machine learning-based techniques.

**Table 3:** Overview of best-performed algorithms on 5 most-popular public datasets

	UBFC-rPPG^53^		PURE^54^		COHFACE^55^		VIPL-HR^56^		MAHNOB-HCI^57^	
Article	MAE	RMSE	MAE	RMSE	MAE	RMSE	MAE	RMSE	MAE	RMSE
Qian W et al.^20^	0.17	0.41	0.37	0.68			4.36	6.92		
Zhao P. et al.^14^	0.12	0.35	0.08	0.29						
Hao Lu et al.^12^	0.44	0.67	0.82	1.31			4.93	7.68		
Si-Qi Liu et al.^65^	0.31	0.98	0.18	0.41	0.64	1.89			3.13	3.97
Yaran Duan et al.^62^	2.78	3.35	2.76	4.15			2.77	3.87	3.45	4.17

Performance metrics: MAE, RMSE. Datasets: UBFC-rPPG, PURE, COHFACE, VIPL-HR, MAHNOB-HCI.

It is important to note that these approaches have been tested on a variety of datasets with differing levels of complexity. This variability can result in lower performance on datasets containing motion artifacts, particularly in terms of MAE and RMSE. Nonetheless, the findings consistently show that recent machine learning methods with multiple ROIs outperform earlier techniques across all publicly available datasets.

### Influence of light and motions to accuracy

Several well-known challenges complicate heart rate estimation via remote photoplethysmography (rPPG). Light reflections on the subject’s facial skin can cause inaccurate signal readings, leading to errors in heart rate predictions. Additionally, poor video quality-resulting from factors such as the subject’s distance from the camera, insufficient lighting, improper face detection, occluded facial regions, and excessive movement-can significantly impact precision.

Many datasets used in rPPG research were collected in controlled environments^53,55^, predominantly featuring young to middle-aged white male subjects. As a result, models trained on these datasets often struggle to accurately predict heart rates across diverse populations or under real-world conditions. In contrast, the VIPL dataset^56^ presents a more challenging scenario by incorporating various conditions, including head movements, rotations, speech, facial expressions, and both low and high lighting levels. These factors contribute to the dataset’s complexity and are evident in performance analyses, underscoring the need for models capable of handling diverse environmental and demographic variables.

Across the reviewed studies, commonalities in data exclusion practices were observed, particularly in frames where face-detection algorithms (e.g., Dlib, MediaPipe, Viola-Jones) failed to accurately capture the face. Additionally, most studies excluded regions such as the eyes, mouth, and areas with facial hair from analysis. When certain facial patches became obscured due to head rotation or other factors, they were often excluded from rPPG estimation or substituted with their symmetrical counterparts to maintain data integrity.

## Discussion

The objective of this review was to assess the existing literature on challenges associated with region of interest (ROI) selection for heart rate estimation in remote photoplethysmography (rPPG) algorithms. We conducted a comprehensive search to identify relevant studies, focusing on how the choice and quantity of facial patches influence the performance of rPPG-based heart rate estimation.

This review identifies several limitations that warrant consideration. First, despite conducting an extensive search across PubMed, IEEE Xplore, and Embase, some relevant studies may have been inadvertently missed. Second, certain studies included in this review did not specify details such as the exact number of ROIs analyzed, the datasets used, or the performance evaluation methods applied. These gaps may affect the completeness of the presented data and could influence the interpretation of results. Although these limitations do not detract from the overall value of this review, they highlight the need for future research to provide more comprehensive reporting to deepen insights into this field.

As illustrated in Fig. 4, using a greater number of ROIs generally improves estimation quality, although it remains unclear whether additional patches reduce error or simply add noise. While the diversity of algorithms and ROI selection strategies complicates establishing a definitive trend, our findings suggest a potential negative correlation between the number of ROIs and error metrics, such as Mean Absolute Error (MAE) and Root Mean Square Error (RMSE). However, we recommend conducting further studies with a larger number of ROIs and across diverse datasets to validate or refute the presence of a linear relationship. Nevertheless, findings suggest that using more than four ROIs often enhances accuracy by mitigating noise. Conversely, an excessive number of patches (e.g., more than 100) may introduce additional noise, potentially diminishing accuracy. So, the error for 6 ROIs and 60 ROIs is relatively the same, however, for a second case we require more timing and computational resources. For optimal results, multiple sub-ROIs from areas like the forehead and cheeks, or combinations thereof, may provide the most reliable outcomes.

Based on this review, we recommend the following directions for future research:

- A key research priority for future studies is to investigate whether dividing the face into additional sub-ROIs enhances rPPG signal quality or inadvertently introduces more noise. Addressing this question could provide critical insights into optimizing ROI segmentation strategies for improved accuracy in heart rate estimation. We suggest performing research on diverse datasets by consistently dividing facial ROIs into sub-ROIs to find an optimal distribution threshold, based on the work of Li et al.^61^.
- Prioritize the selection and segmentation of facial ROIs, with a specific focus on the forehead and cheeks. These areas, particularly the forehead and cheeks, can be further divided into smaller sub-ROIs, as studies have shown that increasing the number of well-defined patches improves signal quality and enhances HR estimation accuracy, particularly under challenging conditions like motion artifacts or poor lighting. For instance, recent research utilizing more than three ROIs^19^ or employing up to 2^*n*^ − 1 ROI combinations^20,21^ has consistently reported lower error rates on benchmark datasets. This highlights the potential of sub-ROI segmentation to improve robustness in real-world applications, though further validation is needed to confirm these findings across diverse datasets.
- Improve rPPG signal quality by dynamic ROIs selection and enhancement based on SQI, SNR, histogram and find an optimal threshold value to all those parameters, similar to 0.293 value for SQI^5^. Additionally, resample shared datasets to overcome limited sample sizes and model overfitting.
- Increase the diversity of testing and validation datasets by including more female subjects from varied age groups and racial backgrounds. Additionally, incorporating factors, such as motion, make-up, sweat, facial accessories, distance from camera and light source, multiple subjects and partial facial occlusion, and diverse lighting conditions would contribute to more robust and realistic outcomes in real-world applications.
- Adaptive ROI Orchestration: a promising extension of this review is to quantify and optimize the robustness-versus-flexibility trade-off that arises as the number of ROIs grows. We propose evaluating dynamic ROI-selection frameworks in which the analysis engine continuously ranks each patch by objective quality indicators (e.g., SQI, SNR, or PCC) and, at run time, activates only the subset that exceeds a reliability threshold. Such an adaptive scheduler would:
  1. Fallback for occlusions. Seamlessly switch to alternative patches dynamically when a region is covered by hair, glasses, or head motion. Noise suppression. Exclude low-quality ROIs (based on a single or multiple quality indicators) on a frame-by-frame basis, preventing the dilution of the composite rPPG signal.
  2. Resource awareness. Limit the active ROI set when computational or energy budgets are tight, then re-expand it when resources allow.
  3. Exploring these mechanisms, ideally across datasets with controlled occlusion and motion scenarios, will help establish principled guidelines on when to favor holistic, sparse, or dense ROI configurations and how to transition between them automatically.

## Methods

### Registration and protocol

This review was registered in the PROSPERO database (ID: CRD42024592157) before its initiation. During the preparation of this work, we used ChatGPT (version GPT-4o, OpenAI) to optimize the readability. After using this tool, authors reviewed and edited the content of the manuscript as required and took full responsibility for the publication and its content.

### Literature search and selection criteria

Following PRISMA guidelines for systematic reviews, we conducted a comprehensive literature search across IEEE Xplore, PubMed, and Embase, focusing on studies published from January 2014 to October 2024 to capture recent advancements in heart rate (HR) estimation via remote photoplethysmography (rPPG). The search terms included ‘ROI,’ ‘region of interest,’ ‘patch,’ ‘video,’ ‘image,’ ‘camera,’ ‘smartphone,’ ‘rPPG,’ ‘remote photoplethysmogram,’ ‘transdermal optical imaging,’ ‘TOI,’ ‘heart rate,’ ‘HR,’ ‘blood pressure,’ ‘face,’ and ‘facial,’ with Boolean operators applied to expand the scope of relevant studies. The initial search was conducted by one author (M.B.), with a second author (M.E.) independently verifying the results to ensure accuracy. Studies were included if they reported on HR or BP estimation using facial regions captured by a standard RGB camera. Exclusion criteria encompassed duplicate publications, inaccessible articles (i.e., those without full-text availability), studies irrelevant to HR or BP prediction, review articles, theoretical papers, studies using non-RGB cameras (e.g., NIR cameras), and studies employing non-contactless devices or analyzing non-facial regions.

### Data analysis and statistical approach

In this review, we systematically analyzed the impact of ROI selection on heart rate estimation accuracy by assessing algorithmic errors, specifically Mean Absolute Error (MAE) and Root Mean Squared Error (RMSE), across publicly available datasets. Given the variety of datasets used for training and testing, establishing a definitive optimal approach is challenging. However, statistical evidence indicates that recent machine learning-based methods utilizing combinations of multiple ROIs generally achieve superior performance.

To enhance the selection of ROIs beyond statistical data, we recommend incorporating a Signal Quality Index (SQI) analysis for each ROI’s rPPG signal. By applying a threshold value of 0.293, ROIs and corresponding signals with excessive noise can be excluded. This approach ensures that only high-quality signals contribute to heart rate (HR) detection, thereby improving the overall accuracy and robustness of the measurements.

## Supplementary information

Supplementary information

## Data Availability

No datasets were generated or analyzed during the current study.

## Code availability

No custom code or software was developed for this study. All analyses were conducted using publicly available tools and standard computational methods.
