# Supplementary material for: The role of face regions in remote photoplethysmography for contactless heart rate monitoring
Source: NPJ Digit Med. 2025 Jul 26;8:479. doi: 10.1038/s41746-025-01814-9 (PMC12297079; doi:10.1038/s41746-025-01814-9)
Supplement: Supplementary file 1 — Supplementary information [file 41746_2025_1814_MOESM1_ESM.pdf]

Supplementary Information: number of ROIs to performance for COHFACE, MANHOB-HCI and PURE datasets

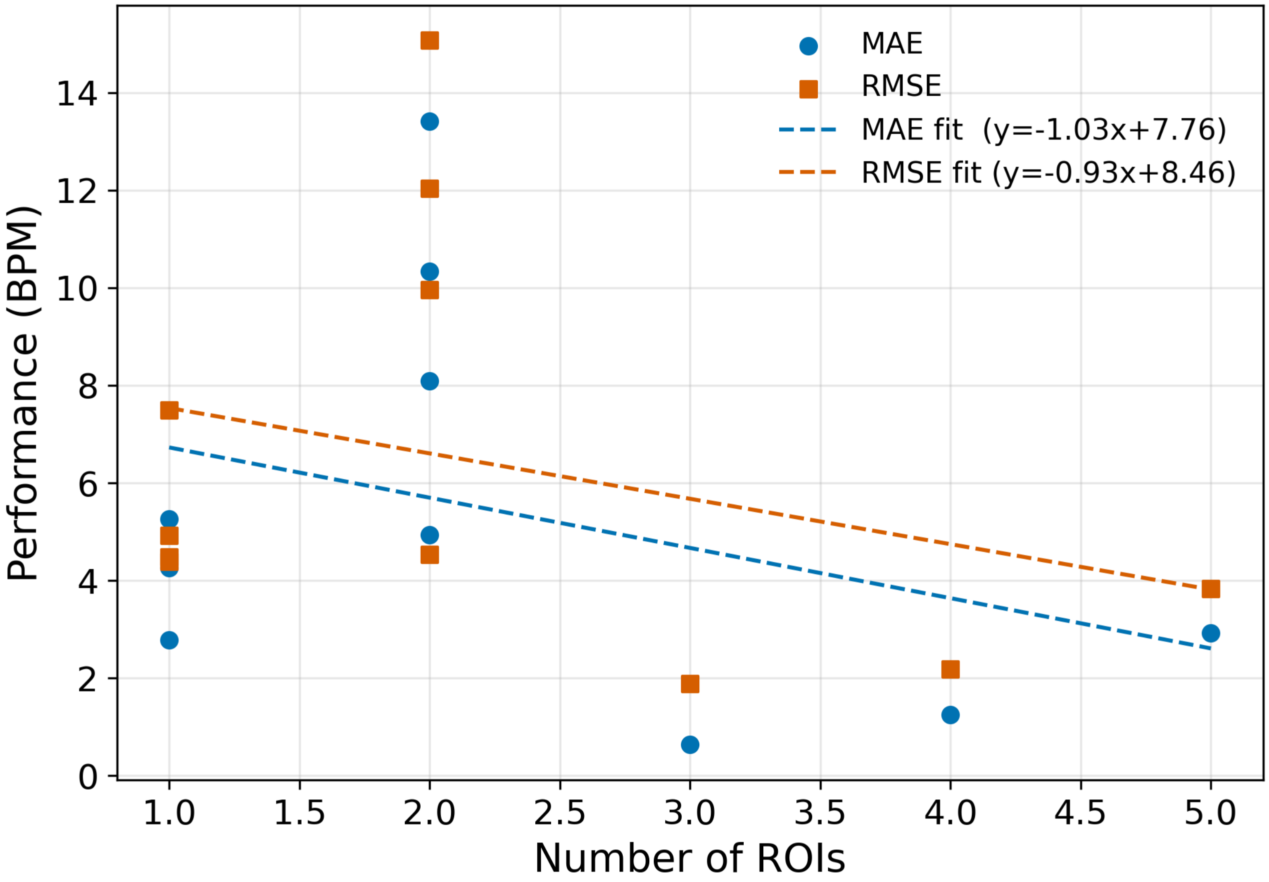

Figure S1. Supplementary Figure S1. COHFACE dataset: number of ROIs to performance. R^2 values indicate a modest linear fit (0.114 for MAE and 0.128 for RMSE).

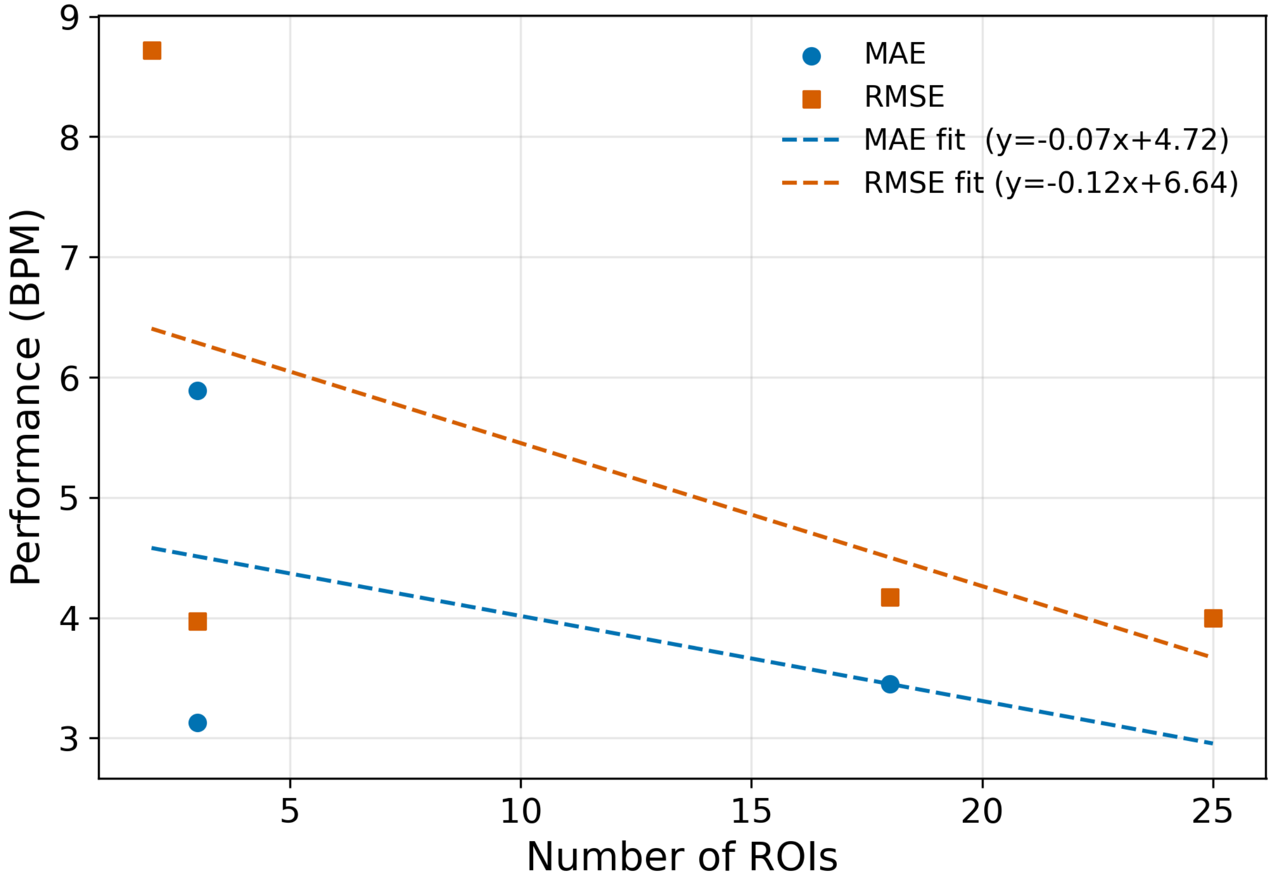

Figure S2. Supplementary Figure S2. MANHOB-HCI dataset: number of ROIs to performance. R^2 values indicate a modest linear fit (0.16 for MAE and 0.33 for RMSE).

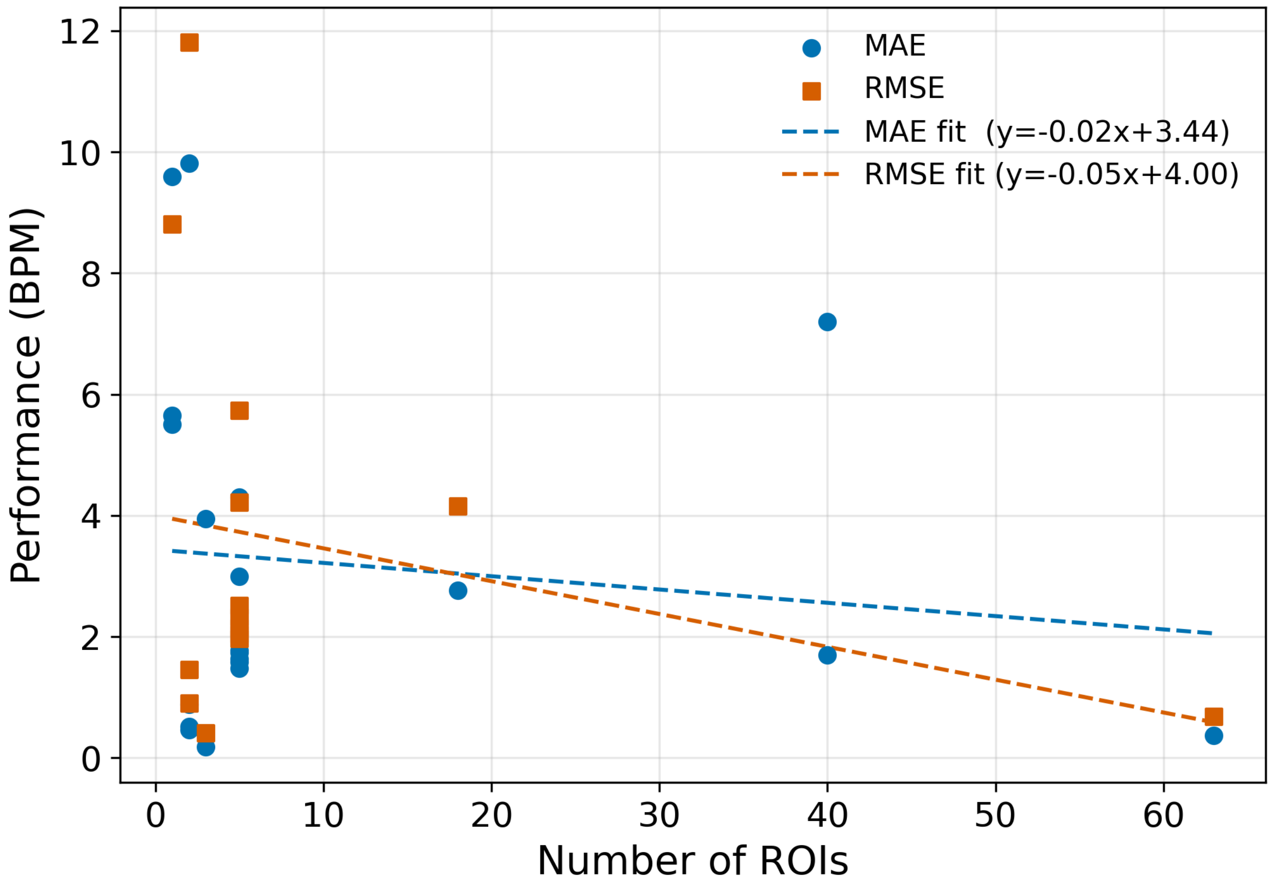

Figure S3. Supplementary Figure S3. PURE dataset: number of ROIs to performance. R^2 values indicate a weak linear fit (0.016 for MAE and 0.070 for RMSE).
